# On analysing clinical trial data using the change from baseline

**DOI:** 10.1177/14653125211059544

**Published:** 2021-12-07

**Authors:** Spyridon N. Papageorgiou

**Affiliations:** Clinic of Orthodontics and Pediatric Dentistry, Center of Dental Medicine, University of Zurich, Zurich, Switzerland

## Theoretical scenario

For this theoretical scenario, the effect of reinforcing mandibular anchorage with temporary anchorage devices during correction of Class II malocclusion with fixed functional appliances will be analysed. In particular, the effect of anchorage reinforcement on reducing lower incisor inclination (1i-ML; in degrees) will be assessed. The studies identified from two recently published systematic reviews (Al-Dboush et al., 2021; Huang et al., 2021) are pooled here and their data re-analysed. Some studies initially included in the original systematic reviews are excluded however, since they measured inclination of the lower incisors relative to the NB line and not to the mandibular line or did not report complete descriptive statistics pre-treatment, post-treatment, and in terms of treatment-induced changes (post minus pre).

In the end, seven included papers remained (2 randomised trials and 5 non-randomised studies), which compared a Class II group treated with skeletally anchored fixed functional appliances (experimental group) and a Class II group treated with conventional fixed functional appliances (anchored on the teeth; control group). The results of these studies have been here re-analysed with conventional random-effects (REML) meta-analyses in terms of Mean Differences (MDs) with their 95% Confidence Intervals (CIs) using (a) the post-treatment measurements and (b) the treatment-induced changes (post minus pre). However, pre-treatment differences among the compared groups (i.e. if the two study groups are not adequately comparable at baseline) can influence the observed treatment effects. Therefore, meta-regression of pre-treatment differences on the observed treatment effects were performed. All analyses were done according to current guidelines with a P<0.05 considered significant for the meta-analyses and a P<0.10 considered significant for the meta-regressions.

## Which of the following statements is correct, if any?

A.  The direction of each study’s effect (whether it favours skeletal reinforced appliances or conventional appliances) is the same in the analysis of Post-Pre increments and in the analysis of Post values.
B.  The magnitude of the pooled treatment effect from the meta-analysis is the same in both analyses.
C.  The P value for the pooled treatment effect from the meta-analysis is either statistically significant (P<0.05) or not (P≥0.05) in both analyses.
D.  Any baseline discrepancies (dissimilarities; imbalances in the covariate of Pre 1i-ML)) between the two compared groups will significantly impact only the analysis of final (Post) values, but not the analysis of treatment changes (Post-Pre).

## Discussion

**Figure 1.**
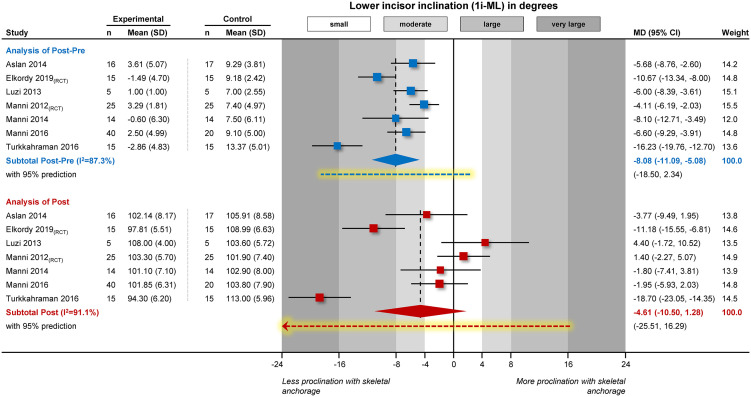
Contour-enhanced forest plot on lower incisor inclination after Class II treatment with fixed functional appliances reinforced with temporary anchorage devices (experimental group) compared to conventional fixed functional appliances (control group). Meta-analysis is performed either using treatment changes (Post-Pre) or finale post-treatment values (Post). CI, confidence interval; MD, mean difference; RCT, randomised clinical trial.

The results of the 2 analyses of the included studies (either using Post-Pre increments or the Post measurements) are illustrated in Figure 1. Even by eyeballing the forest plot it is obvious that the same studies differ considerably in the direction of the treatment effect according to the level of the analysis. In the analysis of treatment changes (Post-Pre), all studies are on the left side of the forest plot, which indicates in all instances a benefit for skeletal reinforcement in terms of reduced lower incisor proclination. On the other side, based on the final (Post) values, for some studies the MDs are on the left side (favour the experimental group), while others are on the right side (favour the control group). We can therefore see that statement A is wrong and the direction of the treatment effect is dependent on what kind of data are analysed.

The results of the two meta-analyses are given apart from Figure 1 also in detail in Table 1. From Figure 1, one can see that there is a big discrepancy at the magnitude of the observed effects between the two analyses. In the analysis of treatment increments (Post-Pre) on Figure 1, most of the studies present a moderate to large benefit for skeletal anchorage and one study presents a very large benefit. In the analysis of post-treatment (Post) values, the majority of the studies show a small / clinically irrelevant effect (are in the white areas of the plot), one shows a large benefit and one a very large benefit. This discrepancy between the two analyses is capitalised in the pooled effect of the meta-analysis: the pooled effect of treatment increments (Post-Pre) indicates an MD of -8.08°, which can be considered a moderate to large benefit from skeletal anchorage (based on its 95% CI). On the other hand, the pooled effect from the post-treatment values (Post) gives an MD of -4.61°. This is almost half the effect of -8.08° seen from treatment increments and spans (based on its 95% CI) from a clinically irrelevant effect from the right or left side of the plot up to a large beneficial effect on the left side. It is therefore clear that the magnitude of the two effects is not similar (statement B is wrong), but also that there is considerably greater imprecision / heterogeneity for the second analysis.

**Table 1.** Meta-analysis of 7 studies comparing lower incisor inclination after Class II treatment with skeletally-anchored fixed functional appliances compared to conventional ones, using either the Post-Pre increment or the Post values.

Analysis	MD (95% CI)	P	I^2^ (95% CI)	95% prediction
Treatment changes (Post-Pre)	-8.08 (-11.09, -5.08)	<0.001	87.3% (65.4%, 96.9%)	-18.5, 2.34
Final values (Post)	-4.61 (-10.5, 1.29)	0.13	91.1% (76.8%, 97.8%)	-25.51, 16.29

CI, confidence interval; MD, mean difference.

Looking at the results of Table 1 it is also obvious that the meta-analysis of treatment changes (Post-Pre) is statistically significant indicates a departure from the null hypothesis of no between-groups difference, whereas the second analysis is compatible with the scenario of no differences between the two treatment groups (P>0.05). This can also be seen from Figure 1, where the red diamond crosses the vertical line of no effect (meaning P>0.05), which means that statement C is also wrong.

**Figure 2.**
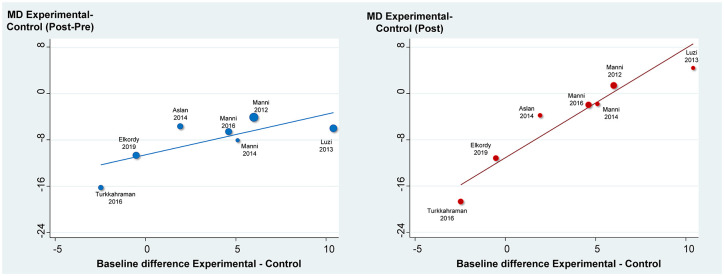
Meta-regressions of baseline (Pre) imbalances in lower incisor inclination between the experimental – control group on the observed treatment effect (MD). Analysis is performed either using treatment changes (Post-Pre) or finale post-treatment values (Post). MD, mean difference.

In order to assess the effect of any baseline discrepancies on the measured outcome, meta-regressions of the baseline (Pre) incisor inclination difference on the treatment effects (MDs) of skeletal reinforcement were run and are seen in Figure 2. In the analysis of treatment changes (Post-Pre) a statistically significant effect was seen (P=0.05 <0.10) with the MDs being on average 0.70° greater (95% CI: 0 to 1.40°) for each ° of baseline discrepancy between experimental – control groups. In the analysis of post-treatment (Post) values again a statistically significant effect was seen (P=0.001) with the MDs being on average 1.89° greater (95% CI: 1.24 to 2.54°) for each ° of baseline discrepancy between experimental – control groups. We see that baseline dissimilarities between the two groups influence more the analysis of the post-treatment values – which is logical, since in this analysis the pre-treatment values are not taken into consideration. However, baseline dissimilarities between the experimental – control groups also influence the analysis of treatment changes (Post-Pre) and therefore statement D is also wrong.

Finally, an interesting observation can be made if one considers the design of the included studies in conjunction with their results. Theoretically, one might expect that in the analysis of randomised trials, the two analysis methods (based on Post-Pre increments or on Post values) would yield similar results, since baseline similarity (or better similar distributions of all covariates) between experimental – control groups is expected. For the first randomised trial, from Elkordy et al. (2019), similar MDs are seen for the two analyses (-10.67° and -11.18°), which is logical, since at baseline, the experimental – control groups are very similar (differ only by 0.52°). On the other hand, this is not the case for the randomised trial of Manni et al. (2012), which has an MD based on treatment changes (Post-Pre) of -4.11° and an MD based on final (Post) values of +1.40° (Figure 1). This might be explained at least to some extent by the fact that at baseline the experimental and control groups differ considerably (by 6.00°; 95% CI: 3.01 to 8.99°). Possible explanations for this might include the small recruited sample of patients that precludes balanced distribution among the two groups (Senn, 2013), a compromised randomisation procedure, or pure chance.

These issues highlight the complexity one often faces when designing and analysing the results of clinical trials that was raised previously (Papageorgiou, 2021) and might potentially be alleviated by the transparent provision of a clinical trial’s dataset (Papageorgiou and Cobourne, 2018) in order to re-analyse data using the post-treatment values and adjusting for pre-treatment imbalances that has been shown to present considerable advantages.
